# Transcriptome Analysis Reveals Long Intergenic Non-Coding RNAs Contributed to Intramuscular Fat Content Differences between Yorkshire and Wei Pigs

**DOI:** 10.3390/ijms21051732

**Published:** 2020-03-03

**Authors:** Qianqian Li, Ziying Huang, Wenjuan Zhao, Mengxun Li, Changchun Li

**Affiliations:** 1 Key Laboratory of Agricultural Animal Genetics, Breeding, and Reproduction of the Ministry of Education and Key Laboratory of Swine Genetics and Breeding of the Ministry of Agriculture, Huazhong Agricultural University, Wuhan 430070, China; lqq101521@163.com (Q.L.); huangziyingtg@163.com (Z.H.); zwj163oyouxiang@163.com (W.Z.); 18699301319@163.com (M.L.); 2 Guangxi Yangxiang Co., Ltd. Production Center, Guigang 537131, China

**Keywords:** lincRNAs, Yorkshire pig, Wei pig, intramuscular fat content, RNA-Seq

## Abstract

Intramuscular fat (IMF) content is closely related to various meat traits, such as tenderness, juiciness, and flavor. The IMF content varies considerably among pig breeds with different genetic backgrounds. Long intergenic non-coding RNAs (lincRNAs) have been widely identified in many species and found to be an important class of regulators that can participate in multiple biological processes. However, the mechanism behind lincRNAs regulation of pig IMF content remains unknown and requires further study. In our study, we identified a total of 156 lincRNAs in the longissimus dorsi muscle of Wei (fat-type) and Yorkshire (lean-type) pigs using previously published data. These identified lincRNAs have shorter transcript length, longer exon length, lower exon number, and lower expression level as compared with protein-coding transcripts. We predicted potential target genes (PTGs) that are potentially regulated by lincRNAs in cis or trans regulation. Gene ontology and pathway analyses indicated that many potential lincRNAs target genes are involved in IMF-related processes or pathways, such as fatty acid catabolic process and adipocytokine signaling pathway. In addition, we analyzed quantitative trait locus (QTL) sites that differentially expressed lincRNAs (DE lincRNAs) between Wei and Yorkshire pigs co-localized. The QTL sites where DE lincRNAs co-localize are mostly related to IMF content. Furthermore, we constructed a co-expressed network between DE lincRNAs and their differentially expressed PTGs (DEPTGs). On the basis of their expression levels, we suggest that many DE lincRNAs can affect IMF development by positively or negatively regulating their PTGs. This study identified and analyzed some lincRNAs- and PTGs-related IMF development of the two pig breeds and provided new insight into research on the roles of lincRNAs in the two types of breeds.

## 1. Introduction

Considerable differences in intramuscular fat (IMF) content have been reported between Chinese indigenous and Western pig breeds due to their varying genetic backgrounds and artificial breeding methods. Yorkshire, a typical lean-type Western breed, has higher growth and lean meat content. By contrast, Wei pig, a typical Chinese indigenous black fat-type breed that is mostly distributed in the southern region of Anhui Province, China, has high IMF content and excellent meat quality [1]. The two pig breeds, which differ substantially in phenotype and exhibit considerable differences in muscle development and IMF content, are good animal models for the identification of differentially expressed genes that contribute to IMF content differences and for determining the molecular mechanisms of these differences between the muscles of Yorkshire and Wei pigs.

IMF content is a key meat quality trait that affects the tenderness, flavor, and juiciness of pork [2], and poor meat quality of modern lean pigs is caused by the significant decrease in IMF content [3]. In addition, studies on the mechanisms of IMF development can promote pork meat quality. Previous studies have shown that certain regulators, such as mesenteric estrogen dependent adipose gene [4], arachidonate 5-lipoxygenase-activating protein [5], and miR-130a [6], are significantly related to porcine IMF content. These regulators focus on microRNA and protein-coding genes. Thus, studies on the roles of long intergenic non-coding RNAs (lincRNAs) in porcine IMF development are still lacking.

LincRNAs are a class of RNA transcripts that are more than 200 nucleotides in length, without protein-coding ability [7], and are located between genes that encode proteins [8]. An increasing number of lincRNAs have been identified through sequencing in recent years, and many studies have indicated that lincRNAs are an important regulator of skeletal muscle growth and development [9]. In addition, a number of studies have shown that certain lincRNAs play important roles in different biological processes and pathways, such as TLR and inflammatory signaling, T cell activation [10], apoptotic process, negative regulation of cell proliferation [11], and actin cytoskeleton reorganization [12]. Many lincRNAs have been identified in mice and humans; however, some of these lincRNAs remain unidentified in pigs [13,14]. At present, the lincRNAs involved in fat deposition or lipid metabolism in pigs have been rarely reported, and an in-depth functional analysis of lincRNAs for IMF development in pigs has not yet been conducted.

In our study, we used the RNA-Seq data published in the National Council for Biotechnology Information (NCBI) to assemble the transcriptome of the longissimus dorsi muscle (LDM) of Yorkshire and Wei pigs [15]. We identified 156 putative lincRNAs and presented the characterization of these identified lincRNAs in the LDM of Yorkshire and Wei pigs. In addition, we performed differential expression analysis to identify differentially expressed lincRNAs (DE lincRNAs) that include two novel lincRNAs and 22 known lincRNAs and differentially expressed protein-coding genes (DEGs) on the basis of expression levels between the two breeds. Then, we mapped the DE lincRNAs onto the QTL database and performed QTL analysis to predict their function. We also predicted the potential target genes (PTGs) of DE lincRNAs using two approaches and performed gene ontology (GO) and pathway analyses of these PTGs. Here, we found that some of these PTGs significantly participated in IMF development-related biological processes. This study not only explores the effects of lincRNAs on IMF development between Yorkshire and Wei pigs, but also provides new insight into the functional analysis of lincRNAs.

## 2. Results

### 2.1. Transcripts Assembly and Identification of LincRNAs

**Figure 1:**
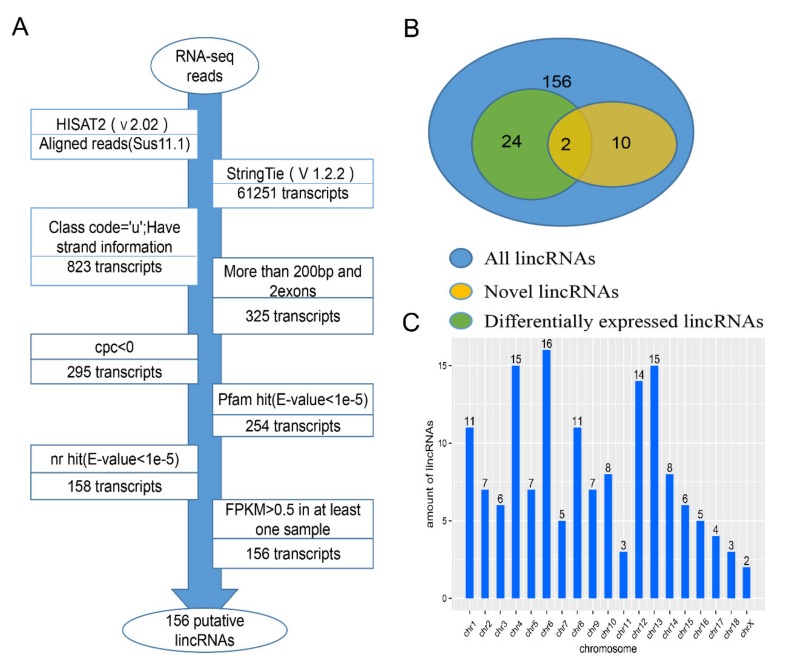
(**A**) Identification pipeline of lincRNAs; (**B**) Venn program of different kinds of lincRNAs; (**C**) distribution of lincRNA on chromosome.

To study the differences in growth and meat quality between Western commercial pigs and Chinese indigenous pigs, we used previously published RNA-Seq data to identify and analyze lincRNAs in the LDM of two types of pigs, Wei and Yorkshire [1].The number of clean reads for the samples was 410.81 million after removing the adaptor and low-quality reads; in addition, 225.11 of 410.81 million were mapped onto pig genome (Sus scrofa 11.1) using HISAT2 (Table 1). Then, the alignments were passed to StringTie for the transcript assembly of each sample. After the assembly of each sample, we merged all the transcripts into a unique transcriptome using StringTie’s merge function [16]. We identified lincRNA following the process shown in Figure 1A. Finally, we found 156 putative lincRNAs, including 144 known and 12 novel lincRNAs, from 823 intergenic transcripts, according to the illustration in Figure 1A. In addition, there were 24 DE LincRNAs, include two novel lincRNAs and 22 known lincRNAs (Figure 1B). The identified putative lincRNAs were distributed on all chromosomes, except for the Y chromosome (Figure 1C).

### 2.2. Characterization of Protein-Coding Transcripts and Identified LincRNAs

**Figure 2:**
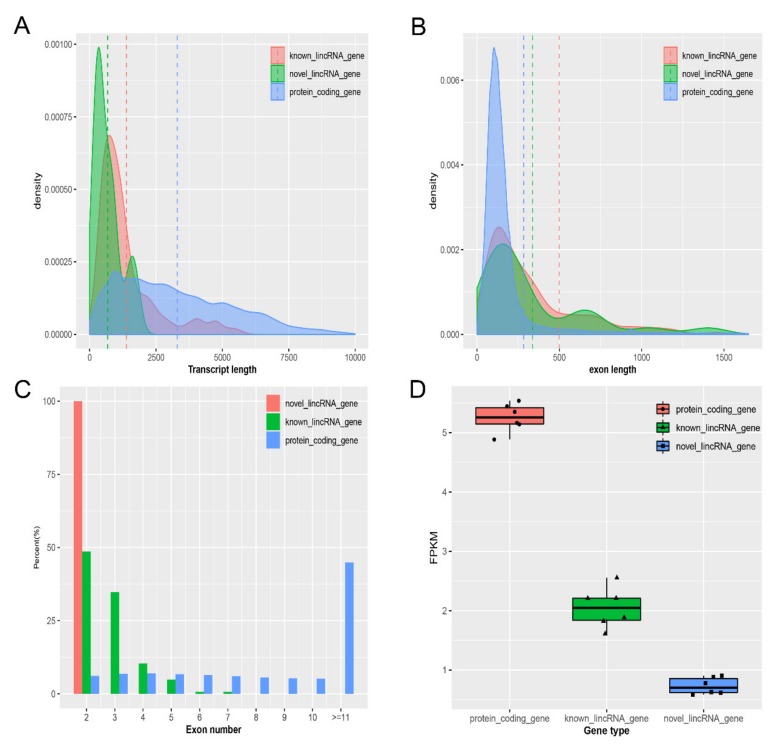
Characteristic of identified lincRNAs. (**A**) Comparison of transcript length; (**B**) comparison of exon length; (**C**) comparison of exon number; (**D**) comparison of expression level.

Previous studies have shown that many differences in features exist between lincRNAs and protein-coding transcripts [17,18]. To verify this result, we analyzed the characteristics, including the transcript length, exon length, exon number, and fragments per kilobase of transcript per million mapped reads (FPKM) of the identified lincRNAs on the basis of the reconstructed transcriptome and, then, compared these characteristics with those of protein-coding transcripts. We then obtained 45,788 protein-coding transcripts corresponding to 24,322 protein-coding genes from the Ensembl pig annotation database and 12,103 known lincRNA transcripts corresponding to 7381 known lincRNA genes from the pig lincRNA annotation file in the domestic animal lincRNA database (ALDB). In our study, the average transcript length of novel lincRNAs was 677 bp, and those of known lincRNAs and protein-coding transcripts were 1382 bp and 3296 bp, respectively. From these results, we concluded that the average transcript length of protein-coding transcripts was longer than those of known and novel lincRNAs (Figure 2A). In addition, the average exon lengths of novel lincRNAs, known lincRNAs, and protein-coding transcripts were 338, 499, and 283 bp, respectively. These findings showed that the average exon length of protein-coding transcripts was shorter than those of novel and known lincRNAs; however, the average exon length of known lincRNAs was longer than that of novel lincRNAs (Figure 2B). Simultaneously, the average exon number of known lincRNAs (2.7) was similar to that of novel lincRNAs (2). Meanwhile, protein-coding transcripts (11.6) were more than known and novel lincRNAs (Figure 2C). In addition, the average expression levels of novel lincRNAs, known lincRNAs, and protein-coding transcripts were 0.7, 2.0, and 5.2 FPKM, respectively. Our results showed that the average expression level of protein-coding transcripts was higher than that of lincRNAs (Figure 2D). By contrast, lincRNAs had shorter transcript length, longer exon length, fewer exon number, and lower expression level than protein-coding transcripts. These results are consistent with previous reports [19,20].

### 2.3. Differential Expression Analysis of LincRNAs and Protein-Coding Genes

**Figure 3:**
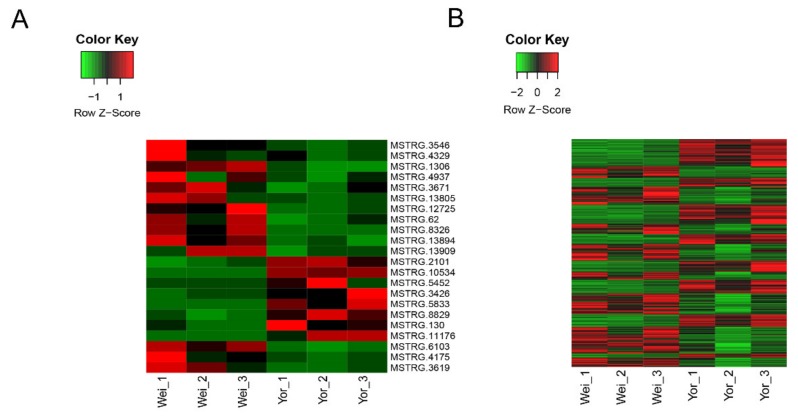
Differential expression analysis of lincRNAs and protein-coding genes in two pig breeds, (Wei) Wei pig and (Yor) Yorkshire pig. (**A**) 22 differentially expressed lincRNAs between the Wei group and Yorkshire group; (**B**) 591 differentially expressed protein-coding genes between the Wei group and Yorkshire group.

We conducted differential expression analysis between Wei and Yorkshire pigs to investigate the function of lincRNAs based on expression level. We detected 22 DE lincRNAs between the two breeds; among which, 14 were upregulated and eight were downregulated DE lincRNAs in Wei pigs as compared with in Yorkshire pigs (Figure 3A). Simultaneously, we obtained 591 DEGs; among which, 306 were upregulated and 285 were downregulated in the Wei group as compared with in the Yorkshire group (Figure 3B).

### 2.4. Association Analysis Between QTL Sites and DE LincRNAs Location

**Figure 4:**
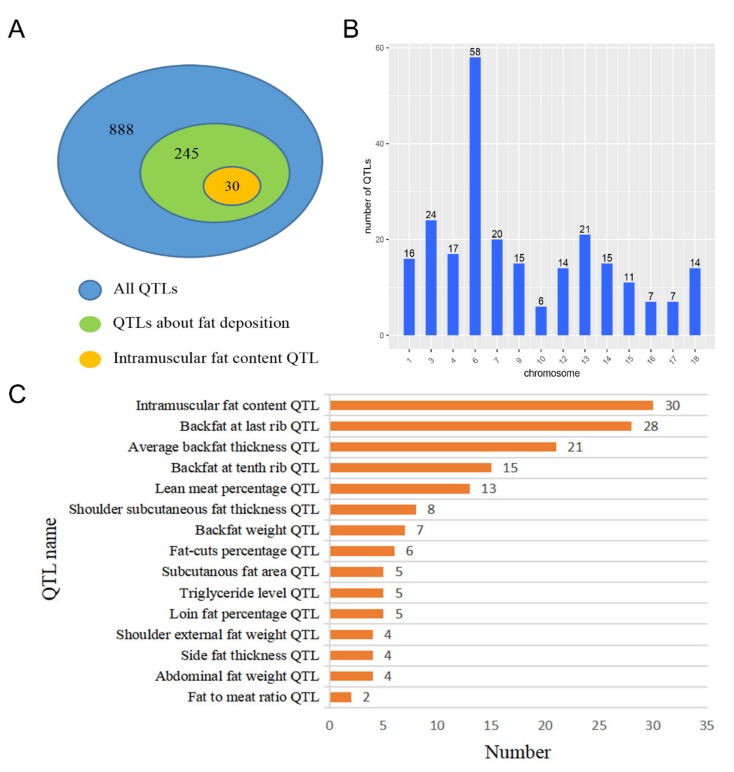
Quantitative trait locus analysis of DE lincRNAs. (**A**) The number distribution of quantitative trait loci (QTLs) associated with fat deposition and all QTLs; (**B**) the chromosome distribution of QTLs associated with fat deposition; (**C**) the number of QTLs associated with fat deposition.

To predict the function of DE lincRNAs, we mapped DE lincRNAs onto the QTL database and performed QTL analysis. The QTL analysis results showed that 22 DE lincRNAs were located in 888 QTLs (Supplementary Table S1). Approximately 27.5% (245/888) QTLs were associated with fat deposition; among which, approximately 12.2% (30/245) were IMF content QTLs (Figure 4A). Through the distribution of QTLs on chromosomes, we found that 245 QTLs related to fat deposition were distributed in 1, 3, 4, 6, 7, 9, 10, 12, 13, 14, 15, 16, 17, and 18 chromosomes (Figure 4B). Moreover, most of these QTLs were associated with backfat, for example, 28 of 245 QTLs were backfat at last rib QTLs, 21 were average backfat thickness QTLs, and 15 were backfat at last rib QTLs. However, the largest number of QTLs associated with fat deposition was IMF content QTLs (Figure 4C).

### 2.5. Prediction of Target Genes of DE LincRNAs

Previous studies have shown that lincRNAs can regulate gene expression in certain ways, and lincRNAs has no fixed mode of action on target genes; thus, gene regulation can occur in cis or trans modes [21]. In our study, we predicted the PTGs of lincRNAs using two analysis methods (discussed in the Materials and Methods Section of this paper). First, we predicted cis-regulated PTGs and obtained 11 PTGs that corresponded to seven DE lincRNAs; meanwhile, six of the 11 PTGs were differentially expressed between two groups. We performed correlation analysis between DE lincRNAs and its neighboring protein-coding genes (< 100 kb) on the basis of their expression levels. The results showed that seven DE lincRNAs were significantly positively correlated with 10 of 11 PTGs. Meanwhile, DEL MSTRG.12725 was negatively correlated with *OTUB2* (Table 2). Then, we predicted PTGs via trans mode and obtained 3255 PTGs of 22 DE lincRNAs; 419 of the 3255 PTGs that corresponded to 21 DE lincRNAs were differentially expressed between two groups. For each DEL, the number of differentially expressed PTGs (DEPTGs) was considerably different. For example, lincRNA MSTRG.10534 had 86 DEPTGs, while lincRNA MSTRG.130 had four4 DEPTGs. LincRNA MSTRG.6103 had 82 DEPTGs, followed by MSTRG.13909, had five DEPTGs. In addition, 20 of 21 DE lincRNAs upregulated most of their DEPTGs, and only 1 DEL (MSTRG.1306) downregulated the majority of its DEPTGs (Table 3).

### 2.6. Functional Enrichment Analysis of the PTGs of DE LincRNAs

**Figure 5:**
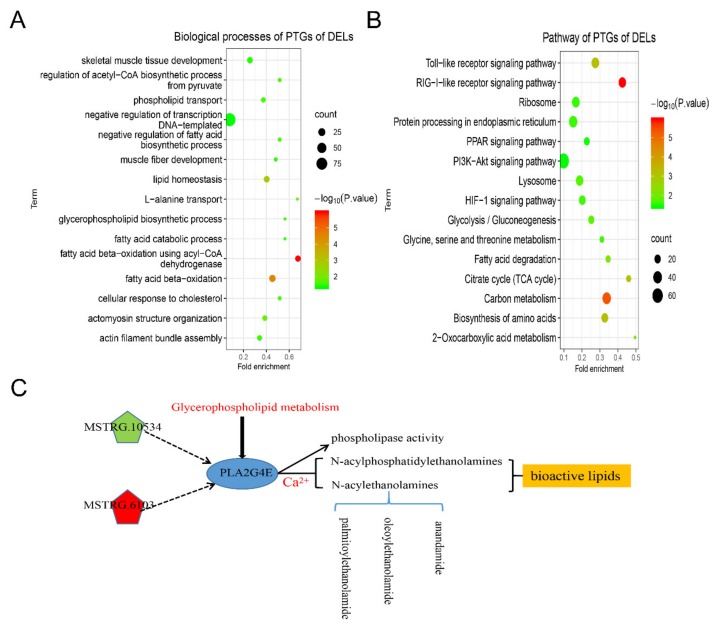
Gene ontology and pathway analysis of the potential target genes (PTGs) of DE lincRNAs. (**A**) Biological processes of PTGs of DE lincRNAs; (**B**) pathways of PTGs of DE lincRNAs; (**C**) the interaction of DEPTGs and DE lincRNAs. The green pentagon represents that the DEL is downregulated in Wei pigs, the red pentagon represents upregulation in Wei pigs, and the blue circle indicates DEPTG.

To predict the functions of the identified lincRNAs and annotate the biological functions of the 3255 PTGs of DE lincRNAs, GO and KEGG enrichment analyses were conducted in our present study. The DAVID results showed that 1425 of the 3225 PTGs were significantly involved in 130 biological processes (*p* < 0.05), mostly in lipid metabolism-related biological processes, such as glycerophospholipid biosynthetic process, fatty acid catabolic process, and negative regulation of fatty acid biosynthetic process (Figure 5A and Supplementary Table S2). In addition, 385 PTGs significantly participated in 29 pathways, including fatty acid degradation, PPAR signaling pathway, and PI3K-Akt signaling pathway (*p* < 0.05) (Figure 5B and Supplementary Table S2). In particular, several PTGs that participated in glycerophospholipid metabolism were highlighted, and the *PLA2G4E* gene participated in this pathway. In addition, the expression level of DEL MSTRG.1306 was upregulated in the Wei group. By contrast, MSTRG.10534 and *PLA2G4E* were downregulated in the Wei group as compared with the Yorkshire group. Furthermore, the expression level of *PLA2G4E* was significantly positively correlated with that of MSTRG.10534 but was significantly negatively correlated with that of MSTRG.1306. The two DE lincRNAs affected lipid metabolism by positively or negatively regulating *PLA2G4E* (Figure 5C).

### 2.7. Expression Regulation Analysis of DE LincRNAs and their DEPTGs

**Figure 6:**
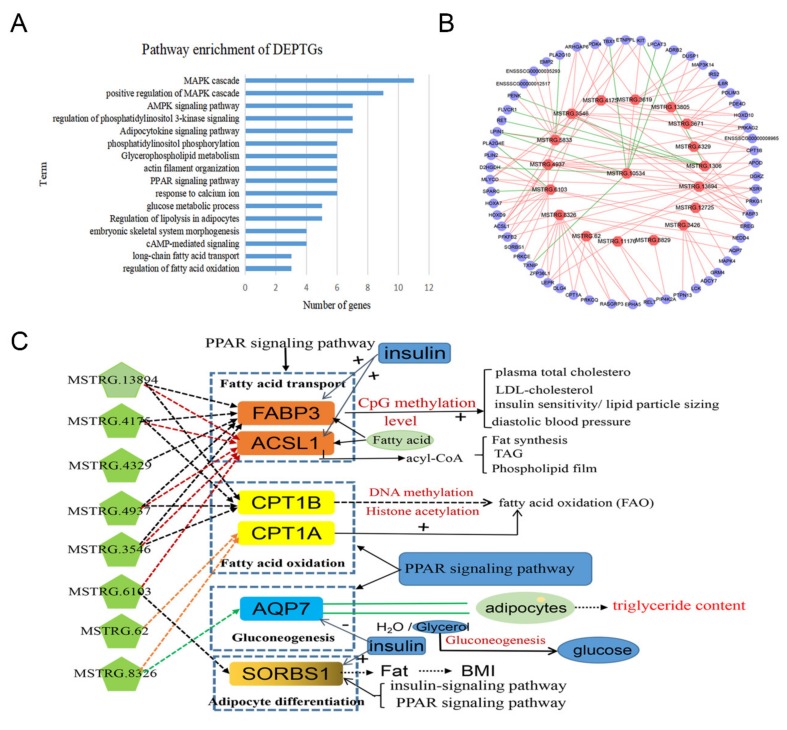
Expression regulation analysis of DE lincRNAs and their DEPTGs. (**A**) Gene ontology and pathway analysis of DEPTGs of DE lincRNAs; (**B**) co-expression network of DEPTGs and DE lincRNAs enriched in lipid metabolism pathways. Pink hexagons represent DE lincRNAs, purple circles represent DEPTGs, a red edge indicates that DE lincRNAs upregulate DEPTGs, and a green edge indicates that DE lincRNAs downregulate DEPTGs; (**C**) the interaction of the major DEPTGs of DE lincRNAs with the lipid metabolism in related pathways, “+” represents positive correlation, “-” represents negative correlation.

To explore the function of DE lincRNAs and understand the expression relationships between lincRNAs and their DEPTGs, we analyzed the expression regulation features of lincRNAs and their DEPTGs. We performed GO and KEGG enrichment analyses of 419 DEPTGs, and the DAVID results showed that 59 DEPTGs were involved in lipid metabolism-related pathways or biological processes (*p* < 0.05), such as adipocytokine signaling pathway and glycerophospholipid metabolism (Figure 6A and Supplementary Table S3). Then, we constructed the lipid metabolism-related DEL-DEPTG co-expression network using Cytoscape_3.6.1 (Institute for Systems Biology, Seattle, Washington, USA) and found that 18 DE lincRNAs exhibit a high degree of co-existing relationship with 59 DEPTGs. In addition, 33 of the 59 DEPTGs were regulated by two or more lincRNAs (Figure 6B and Supplementary Table S4). Furthermore, the regulatory mechanisms between DE lincRNAs and their DEPTGs were complicated.

To further understand the function of DE lincRNAs, we selected two pathways (insulin and PPAR signaling pathways) related to lipid metabolism from all the results of the KEGG enrichment analysis. Six DEPTGs were involved in the two pathways, namely, *AQP7*, *FABP3*, *CPT1B*, *ACSL1*, *SORBS1*, and *CPT1A*. The six DEPTGs and eight DE lincRNAs were upregulated in Wei pigs as compared with in Yorkshire pigs (Figure 6C). In both pathways, eight DE lincRNAs were positively correlated with one or more of the six DEPTGs. *FABP3* and *ACSL1* were associated with fatty acid transport [22,23], and *ACSL1* activated fatty acids and catalyze fatty acids to form acyl-CoA. In addition, acyl-CoA was involved in various metabolic pathways, including the synthesis of triacylglycerols, synthetic phospholipid membranes, and fat synthesis [24]. *CPT1A* and *CPT1B* were involve in fatty acid oxidation [25]. *AQP7* and *SORBS1* are related to gluconeogenesis and adipocyte differentiation, respectively [26,27,28]. Insulin is a crucial hormone in fat synthesis that can positively regulate *ACSL1*, *FABP3*, and *SORBS1* and negatively regulate *AQP7*. These DEPTGs regulated fat metabolism through the mechanism shown in Figure 6C.

### 2.8. Correlation Validation of LincRNAs and their PTGs by RT-qPCR

**Figure 7:**
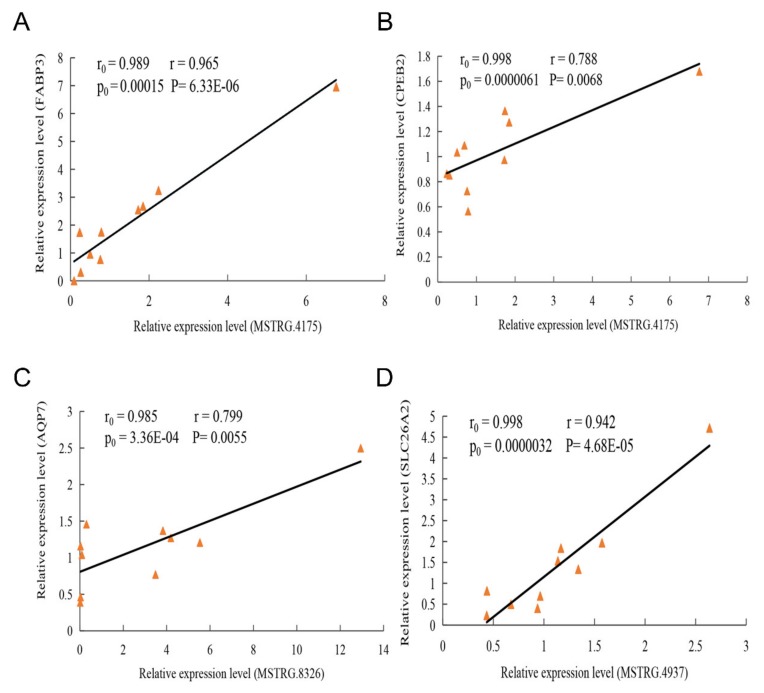
Linear regression of DEL and their DEPTG expression. The r0 and p0 represent the Pearson correlation coefficient and *p*-value of each pair of DE LincRNA and its DEPTG in 6 samples (3 for Yorkshire group, and 3 for Wei group, respectively; while r and P represent verification in 10 samples. (**A**) MSTRG.4175 vs. *FABP3*; (**B**) MSTRG.4175 vs. *CPEB2*; (**C**) MSTRG.8326 vs. *AQP7*; (**D**) MSTRG.4937 vs. *SLC26A2*.

On the basis of their expression levels, we predicted 419 DEPTGs that corresponded to 21 DE lincRNAs in this study. To confirm this result, we randomly selected three DEL genes (Supplementary Table S5) and their DEPTGs for RT-qPCR (MSTRG.4175 vs. *FABP3* and *CPEB2*, MSTRG.4937 vs. *SLC26A2*, and MSTRG.8326 vs. *AQP7*). The results showed that the Pearson correlation coefficients of MSTRG.4175 and its DEPTGs (*FABP3* and *CPEB2*) were 0.965 and 0.788, respectively. Meanwhile, that of MSTRG.4937 and its DEPTG (*SLC26A2*) was 0.799. The correlation coefficients of the four pairs of genes were greater than 0.78, and the *p*-value was less than 0.01 (Figure 7). Meanwhile, the Pearson correlation coefficients of the four pairs of genes obtained in the RNA-Seq were greater than 0.95, and the *p*-value obtained in the RNA-Seq was less than 0.001. The experimental results of the RT-qPCR showed that the two datasets exhibit good consistency.

## 3. Discussion

Pigs are not only important agricultural economic animals, but have also become important biomedical experimental models due to their physiological functions and anatomical structures that are similar to humans [29,30]. Previous studies have shown a large number of lincRNAs in the genomes of mammals, and the number of lincRNAs can be equal or greater than that of protein-coding genes [31,32], and some of lincRNAs have been suggested to play a regulatory role in various biological processes. In addition, the size of the pig genome is essentially the same as that of mice and humans, however, many lincRNAs remain undiscovered in pigs, and several lincRNAs associated with IMF development in pigs are still unidentified. In this study, we used previously published RNA-Seq data to identify and analyze lincRNAs of the LDM of two pig breeds [1]. Here, we identified 156 lincRNAs (144 known and 12 novel lincRNAs) in LDM using our previously published lincRNA identification pipeline [33]. The 12 novel lincRNAs enrich the annotation of pig lincRNA and provide new insight into research on the evolution of lincRNA in pigs.

In this study, we found that lincRNAs exhibits typical features, such as shorter transcript length, fewer exon number, longer exon length, and lower expression level as compared with protein-coding transcripts. The result showed that the features of lincRNAs were consistent with those reported in previous studies [17,20]. In addition, Cabili and Cole et al. proved that lincRNAs have generally higher tissue specificity as compared with protein-coding genes [31]. Thus, we inferred that some lincRNAs identified in LDM could be largely related to muscle growth because of the higher tissue specificity of lincRNAs. Although muscle is an important metabolic tissue in pigs and involved in diverse muscle developmental contexts, such as lipid metabolism and muscle growth, in this study, we provided considerable attention to lincRNAs potentially related to IMF development. The results of the QTL analysis of DE lincRNAs showed that DE lincRNAs were mostly located in IMF content QTL, further proving our speculation in certain aspects.

Exploring lincRNA functions is difficult because lincRNAs have a low expression level and many lincRNA types remain unknown. Previous studies have shown that lincRNAs can regulate gene expression in certain ways, including cis and trans [34,35]. Hong and Kwon used genomic analysis to identify protein-coding genes near (≤ 10 kb) testis-specific lincRNAs to predict the functions of lincRNAs [36]. Zhang and Chen predicted 1061 cis-regulated and 782 trans-regulated targets of DE lincRNAs in their study to explore the DE lincRNAs functions [37]. In our study, we identified 11 cis-regulated PTGs of DE lincRNAs between the Wei and Yorkshire groups, and we found that lincRNAs and their neighbor genes (< 100 kb) exhibited a strong correlation. The expression levels of lincRNA MSTRG.2101 and its PTG calcium voltage-gated channel auxiliary subunit gamma 4 (*CACNG4*) were significant upregulated in Yorkshire pigs (lean-type) as compared with in Wei pigs (fat-type). *CACNG4* can participate in MAPK signaling, organism-specific biosystem pathway, and muscle contraction; in addition, *CACNG4* in homozygote state can decrease adipose tissue accumulation [38]. From these results, we speculated that lincRNA MSTRG.2101 were associated with the lower IMF content and backfat thickness by positively regulating *CACNG4* expression in the Yorkshire group (lean type). Thus, we hypothesized that some lincRNAs can participate in IMF development by regulating their protein-coding neighbor genes. However, the regulatory mechanism by which individual lincRNA regulates its neighbor genes is worthy of further research.

In the study of Xiao et al., they analyzed the functions of lincRNAs on the basis of the co-expressed the lncRNA–mRNA relationship [39]. Here, we performed an analysis of co-expressed lincRNAs and mRNAs and identified 3255 trans-regulated PTGs of DEL between Wei and Yorkshire pigs. We mainly focused on the lipid metabolic processes that these PTGs participated in. In this study, some GO terms and pathways, such as fatty acid catabolic process, glycerophospholipid biosynthetic process, fatty acid degradation, and the TCA cycle, were directly involved in lipid metabolism in accordance with the enrichment results of PTGs, which could account for the higher IMF content in Wei pigs as compared with in Yorkshire pigs. Furthermore, we constructed a pathway map between DE lincRNAs and their PTGs (Figure 5C). The *PLA2G4E* gene was reported to be highly expressed in visceral adipose tissue in colorectal cancer patients and participated in lipid metabolism [40]. Ohto et al. [41] demonstrated that phospholipase activity can be decreased by recombinant *PLA2G4E*. Ogura et al. [42] indicated that *PLA2G4E* can generate and regulate bioactive lipids by mobilizing intracellular calcium in mammalian cells, including N-acyl phosphatidylethanolamines and N-acylethanolamines. In addition, N-acylethanolamines also included palmitoylethanolamide, oleoylethanolamide, and anandamide. Piomelli et al. reported that oleoylethanolamide exhibited an anorexic action through its combination with PPAR-α [43]. In addition, the disruption of the *PLA2G4E*-regulated pathway can cause obesity [44]. Thus, we speculated that MSTRG.10534 promotes phospholipid decomposition and decreases fat accumulation in the LDM of Yorkshire pigs by upregulating *PLA2G4E* expression. Meanwhile, MSTRG.6103 promotes fat deposition by decreasing the expression level of *PLA2G4E* in Wei pigs. Thus, we inferred that DE lincRNAs can contribute to the differences in IMF development between Yorkshire and Wei pigs by regulating their PTGs.

We also analyzed the regulation relationships between DE lincRNAs and their DEPTGs on the basis of their expression levels. We hypothesized that the regulatory mechanisms between DE lincRNAs and their DEPTGs are complicated because of the differences in the number of DEPTGs that DE lincRNAs can upregulate or downregulate. From the DAVID result of DEPTGs, some DEPTGs were involved in some biological processes and pathways related to lipid metabolism, which could further explain the differences in IMF content between Wei and Yorkshire pigs. Ethanolamine-phosphate phospholipase (*ETNPPL*), also known as *AGXT2L1*, lincRNA MSTRG.3546, lincRNA MSTRG.4175, and lincRNA MSTRG.4329, and their target gene *ETNPPL* (*AGXT2L1*), were significantly upregulated in the Wei group as compared with in the Yorkshire group. Moreover, Ding et al. found that *AGXT2L1* is a crucial gene in the abnormal fat formation of hepatocellular carcinoma tissue [45]. Thus, we speculated that the aforementioned lincRNAs can increase fat level in the LDM of Wei pigs by upregulating the expression level of *ETNPPL*.

In addition, some DEPTGs of DE lincRNAs were enriched in the PPAR signaling pathway in our study (Figure 6C). Acyl-CoA synthetase long chain family member 1 (*ACSL1*) has been proven to be involved in fat synthesis, which upregulates the differentiation of 3T3-L1 adipocytes in mice [46]. The level of *ACSL1* mRNA in the LDM of Yorkshire pigs have been reported to be significantly lower than two Chinese native pig breeds [47]. These findings are consistent with our results that the expression level of *ACSL1* is higher and can be associated with higher intramuscular fat in Wei pigs as compared with in Yorkshire pigs. Fatty acid binding protein 3 (*FABP3*) is a known lipid-related gene. Li et al. reported that the polymorphisms of the *FABP3* gene were significantly related to porcine IMF content [48]. In addition, the CpG methylation of *FABP3* strongly affects metabolic syndrome and can lead to obesity [49]. Therefore, the increased expression level of *FABP3* in Wei pigs can contribute to higher IMF content in these pigs. Carnitine palmitoyl transferase 1B (*CPT1B*) and *CPT1A* can affect FAO through DNA methylation and histone acetylation, which can influence fat deposition in muscles. Furthermore, aquaporin 7 (*AQP7*) is a channel of water and glycerol in 3T3-L1 adipocytes that exhibits the effect of controlling the accumulation of triglycerides in adipose tissue [50]. In type 2 diabetes, an increased level of *AQP7* protein abundance in skeletal muscles can contribute to excess lipid accumulation in skeletal muscles [51], similar to our results, and the increased expression level of *AQP7* gene can contribute to a higher IMF content in the muscles of Wei pigs. Furthermore, a previous study indicated that sorbin and SH3 domain containing 1 (*SORBS1*) are highly expressed in liver and skeletal muscles [52], and the expression of *SORBS1* in the adipose tissue can influence adiposity in the adipose depots of nondiabetic women [53]. This finding is consistent with our result, and the increased expression level of *SORBS1* in Wei pigs can affect fat deposition in muscles. Thus, the eight DE lincRNAs in Figure 6C can promote IMF content in Wei pigs as compared with in Yorkshire pigs by upregulating their DEPTGs expression level.

## 4. Materials and Methods

### 4.1. Ethics Statement and Data Acquisition

All experiments in this study were performed according to the guidelines of the Key Lab of Agriculture Animal Genetics, Breeding, and Reproduction of Ministry of Education, Animal Care and Use Committee, Wuhan, China (permit HZAUSW2015-0003). Six female pigs of two breeds (Wei pigs, *n* = 3 and Yorkshire pigs, *n* = 3) were reared under similar conditions [15]. All samples were taken from the same part of the longissimus dorsi muscle at the 3rd and 4th last ribs at a similar weight of 90 kg [15]. Six RNA-Seq datasets (Wei, *n* = 3 and Yorkshire, *n* = 3) were downloaded from NCBI Gene Expression Omnibus (GEO) databases with the accession number provided by Xu et al. [15] (Table 1, GEO accession GSE99092). The pig gene annotations files were downloaded from http://ftp.ensemblorg.ebi.ac.uk/pub/release-93/gtf/sus_scrofa/. Moreover, the non-redundant reference sequence (Refseq) NR database was downloaded from ftp://ftp.ncbi.nlm.nih.gov/blast/db/, and the pig lincRNA annotations were derived from http://res.xaut.edu.cn/aldb/download.jsp.

### 4.2. RNA-Seq Reads Mapping and Transcriptome Assembly

The raw reads were evaluated by FastQc (Nanjing Agricultural University, Nanjing, China) to ensure that high-quality data could be obtained [54], and the raw reads were cleaned by filtering the adapter and low-quality reads using Trimmomatic (version 0.36, Nanjing Agricultural University, Nanjing, China) [54]. Then, the high-quality clean reads were mapped to the pig reference genome (Sus scrofa 11.1, http://ftp.ensemblorg.ebi.ac.uk/pub/release-93/fasta/sus_scrofa/dna/) using the HISAT2 version 2.0.1 (Iowa State University, Ames, IA, USA) with the default parameters [16,55,56], and using SAMtools (version 0.1.19,Wellcome Trust Sanger Institute, Wellcome Trust Genome Campus, Cambridge, UK) to sort and convert the SAM files to BAM [16]. Meanwhile, the -G option of StringTie (version 2.0.2, Johns Hopkins University, Baltimore, MD, USA) was used to assemble transcripts for each sample, and we obtained 6 sample GTF files, respectively [16]. Finally, we used the merge tool of the StringTie package to merge the 6 sample GTF files of two groups into a non-redundant transcriptome [16]. The commands used in this method are as follows:

(1) fastqc -o outdir -t threads fastq1 fastq2.
(2) hisat2 -p 8 --dta --known-splicesite-infile splicesites.txt –x genome -1 sample_1_1_clean.fa -2 sample_1_2_clean.fa –S sample_1.sam
(3) stringtie --merge -p 8 -G genome_reference.gtf -o stringtie_merged.gtf stringtie_merge.txt.

### 4.3. Pipeline for LincRNA Identification

We used the following steps according to the laboratory’s previous studies to identify lincRNAs from the nonredundant transcriptome [19], and the main steps are shown as follows (Figure 1A): (1) Filtered those transcripts without ”u” by using gffcompare (Johns Hopkins University, Baltimore, Maryland, USA), and the ”u” represent intergenic transcripts; (2) transcripts with exon number ≥ 2 and the length of transcripts ≥200 were retained; (3) calculated the coding potential of transcripts in both strands by the coding potential calculation (CPC) tool (Tsinghua University, Beijing, China) [57], and retained the transcripts with CPC values < 0 in any strands; (4) we translated the remaining transcripts sequence into six possible protein sequences by Transeq and used HMMER (HHMI Janelia Fam Research Campus, Ashbum, USA) to identify whether these transcripts had a significant hit in the Pfam databases (E-value < 1 × 10^−5^), then, discarded the transcripts that contained any known protein-coding domain [58]; (5) filtered out the transcripts with similarity to known proteins in the NCBI NR and UniRef90 databases (E-value < 1 × 10^−5^) by using BLASTX program (National Center for Biotechnology Information, Bethesda, USA) [59]; (6) estimated the FPKM values of 6 samples, and then any transcripts with FPKM values less than 0.5 in all samples were filtered out.

### 4.4. Comparisons Between LincRNAs and Protein-Coding Transcripts

We used “grep” command to extract the transcripts annotated as “protein-coding” from the pig reference genome file and obtained 45,788 protein-coding transcripts. Meanwhile, we used the “blastn” command to identify the novel and known transcripts. Then, we compared these lincRNAs with these protein-coding transcripts in transcripts length, exon length, exon number, and FPKM. The command used in this method was as follows:

cat pig reference genome file | grep protein-coding > protein-coding file

### 4.5. Analysis of Differentially Expressed LincRNAs and Protein Coding Genes

In order to perform differentially expressed lincRNAs analysis, we used htseq-count (version 0.9.1, European Molecular Biology Laboratory, Heidelberg, BW, Germany), a tool developed with HTSeq that preprocesses RNA-Seq data for differential expression analysis by counting the overlap of reads with genes [60]. Then, we used the DEseq2 tool (Tsinghua University, Beijing, China) in the R package (version 3.4.3) to perform differential expression analysis of six samples between Wei pigs and Yorkshire [61]. We identified the gene with an absolute fold change value greater than 1 and the corrected *p* -value less than 0.05 as a differentially expressed genes between the two groups [62]. The command used in this method was as follows:

htseq-count -s no -f bam sample_1_sorted.bam stringtie_merged.gtf > sample_1.count

### 4.6. QTLs Analysis of DE LincRNAs

We selected DE lincRNAs to perform QTLs analysis and obtained position information of DE lincRNAs from the unique transcriptome file. Then, we downloaded the pig QTLs database from https://www.animalgenome.org/cgi-bin/QTLdb/SS/index. We used BEDTools (version 2.17.0, University of Virginia School of Medicine, Charlottesville, USA) and “intersectBed” command to perform QTLs analysis [63].

### 4.7. Prediction of PTGs of LincRNAs

We know that gene regulation can occur in either cis or trans, therefore, we can make predictions according to different modes of action. Then, we predicted PTGs of lincRNAs in two ways, including cis- and trans-regulation. For PTGs which were potentially regulated by lincRNAs in cis, we found the protein-coding gene located upstream or downstream (< 100 K) nearby lincRNAs [63,64], and the protein-coding genes were obtained by BEDTools version 2.17.0 (version 2.17.0, University of Virginia School of Medicine, Charlottesville, USA) [65], and then we regarded the protein-coding genes as PTGs of lincRNAs. For PTGs which were potentially regulated by lincRNAs in trans, it showed that the function of lincRNAs was not related to the relationship of position of the protein-coding genes, but was related to the co-expressed protein-coding genes. We regarded protein-coding genes as a PTGs of lincRNAs when these distant protein-coding genes were positively or negatively correlated with the expression of lincRNAs, and the absolute Pearson coefficient (r) between each pair of lincRNA and protein-coding genes was ≥ 0.95, and the FDR-adjusted *p*-value was < 0.05 [62,66]. Meanwhile, if the potential target protein-coding gene was differentially expressed between the two groups, we regarded it as a DEPTG of lincRNAs.

### 4.8. Gene Ontology and Pathway Analysis

In order to clear the function of PTGs of lincRNAs, we used the DAVID (National Cancer Institute at Frederick, Frederick, USA) to perform gene ontology and pathway analyses of these PTGs [67]. We needed to covert these potential target protein-coding gene into human homologous genes using BIOMART from Ensembl [68]. The *p*-value of GO and KEGG pathways less than 0.05 were considered statistically significant.

### 4.9. Correlation Validation Between LincRNAs and PTGs by Real-Time Quantitative PCR

To validate the regulation relationship between lincRNAs and their PTGs, we select 10 RNA samples from longissimus dorsi muscle of two pig breeds, including Wei pigs and Yorkshire pigs. All RNA was extracted using Trizol reagent (Invitrogen, Life Technologies, CA, USA), and we used the Agilent 2100 system to measure the sample concentration. Then, we performed cDNA synthesis for lincRNAs and PTGs detection using the RevertAid First Strand cDNA Synthesis Kit (Termo, Wuhan, Cat# k1622). QPCR for lincRNAs and their PTGs’ detection in Roche LightCyler 480 system (Roche, Mannheinm, Germany) was performed using SYBR Green (CWBIO, Beijing, China, CW0957) according to the manufacturer’s instructions. Primers used for RT-qPCR were designed by Primer Premier 5 program (Supplementary Table S6) and the endogenous control gene used 18s rRNA. The QPCR data were calculated using the 2^-Δ^^ΔCT^ method.

## 5. Conclusions

In our study, we identified and analyzed lincRNAs in the LDM of Wei and Yorkshire pigs and found that some lincRNAs can contribute to the differences in IMF development between the two breeds by regulating their PTGs. In addition, functional analysis revealed that many lincRNAs participated in IMF-related processes, especially DE lincRNAs, thereby resulting in the difference of IMF content between Wei and Yorkshire pigs. However, the function and molecular regulatory mechanisms between lincRNAs and their PTGs remain unclear and requires further exploration. Given that many lincRNAs of pigs are still unknown and the role of lincRNAs in pigs has not been fully annotated, this research provides valuable resources for further studies. Nevertheless, our study provides new insight into the discovery and annotation of lincRNAs associated with IMF content in pigs which represent ideal candidates for further exploration.

## Figures and Tables

**Table 1:** Summary of data from RNA-Seq for Wei and Yorkshire pigs.

Sample	Accession Number	Raw Reads	Clean Reads	Mapped Reads	Mapping Ratio	Uniquely Mapping Ratio
Wei_1	SRR5577192	65911108	52272948	27513396	96.78%	52.60%
Wei_2	SRR5577193	65914286	53989652	29869086	95.69%	55.30%
Wei_3	SRR5577194	93927314	75245286	39681060	96.16%	52.70%
Yor_1	SRR5577189	104766230	84176946	46670672	96.78%	55.40%
Yor_2	SRR5577190	72593892	58591238	32183620	96.84%	54.90%
Yor_3	SRR5577191	105590048	86535990	49194174	96.80%	56.80%

**Table 2:** The correlation between DEL and its neighboring protein-coding genes.

DEL	Adjacent Protein-Coding Gene	Pearson Correlation Coefficient	*p*-Value
MSTRG.12725	ENSSSCG00000002469(***OTUB2***)	−0.915706465	0.010358
	ENSSSCG00000039415(***CCDC19***)	0.817455545	0.04694
MSTRG.13894	ENSSSCG00000039986(***RGS8***)	0.950415666	0.003626
MSTRG.2101	ENSSSCG00000037202(***CACNG4***)	0.826596447	0.042496
MSTRG.3671	ENSSSCG00000038948(***ETS***)	0.87248832	0.02335
MSTRG.4937	ENSSSCG00000015981(***HOXD10***)	0.939146854	0.00544
	ENSSSCG00000015986(***HOXD1***)	0.919787616	0.00939
	ENSSSCG00000034741(***HOXD11***)	0.840680905	0.03605
MSTRG.8326	ENSSSCG00000008218(***RNF103***)	0.86271199	0.026978
	ENSSSCG00000035478(***RMND5A***)	0.929580341	0.00726
MSTRG.8829	ENSSSCG00000005970(***SQLE***)	0.965516179	0.001763

**Table 3:** Summary of differentially expressed lincRNAs (DE lincRNAs) and their differentially expressed potential target genes (DEPTGs).

DE lincRNAs		Number		DE lincRNAs		Number	
	DEPTGs	UpRegulated PTGs	DownRegulated PTGs		DEPTGs	UpRegulated PTGs	DownRegulated PTGs
MSTRG.10534	86	58	28	MSTRG.3619	32	32	0
MSTRG.11176	13	13	0	MSTRG.4175	40	40	0
MSTRG.12725	14	12	2	MSTRG.4937	47	47	0
MSTRG.1306	77	27	50	MSTRG.5833	57	49	8
MSTRG.13894	68	66	2	MSTRG.6103	82	70	12
MSTRG.2101	12	11	1	MSTRG.62	25	24	1
MSTRG.3426	32	30	0	MSTRG.8326	54	48	6
MSTRG.3546	64	64	0	MSTRG.8829	10	10	0
MSTRG.130	4	4	0	MSTRG.3671	20	20	0
MSTRG.13805	19	19	0	MSTRG.4329	20	20	0
MSTRG.13909	5	5	0				

## Supplementary Materials

Supplementary materials can be found at https://www.mdpi.com/1422-0067/21/5/1732/s1. Table S1: QTL mapping analysis on DE lincRNAs, Table S2: Gene ontology and pathway analysis of PTGs of DE lincRNAs, Table S3: Gene ontology and pathway analysis of DEPTGs of DE lincRNAs, Table S4:The expression regulation relationship between DEL genes and their DEPTGs, Table S5: Sequence information of lincRNAs used in RT-qPCR, Table S6: The information of seven pairs of RT-qPCR primers.
