# Supplementary material for: Transcriptome Analysis Reveals Long Intergenic Non-Coding RNAs Contributed to Intramuscular Fat Content Differences between Yorkshire and Wei Pigs
Source: Int J Mol Sci. 2020 Mar 3;21(5):1732. doi: 10.3390/ijms21051732 (PMC7084294; doi:10.3390/ijms21051732)
Supplement: Supplementary file 1 [file ijms-21-01732-s001.zip › Supplementary Materials/TableS5-Sequence information of lincRNAs used in RT-qPCR.docx]

MSTRG.4175

GAGTGCAGCCCTGCTGGCCCTCCTTCAGGGCCTCAGCTTTTCTGAGCGTCGGTCGCCGAGCTGCCCCTCGGAAGCGGCTGCTGGTTTTGGCCGAGGCGGCTTTGGGCAGAAGGAAGTGGACGGGTGATTCATTTCATAAATGTTGGTTCCTCCAGAGAACTGAACAGAACCTGCGACCCTGCAGGTGCTTTTCAACAAGTGGATGACTTCCAGGCCGCTCTAGCCAATGAAGATGGAGCAGGGACCCTGAGATAGAAGGCTTCATTTGGCATGACTCAGCCTTGGAGACATCCATCCCCTTGGCCTGTGTTGTTGAGTGCCTGGTTCTGTAAATATTGGCAGTTAGAAACCAGCCTGAGAGCTACCAAGCAATGATCTGGAAGGACGGCATTATCAAGTGCCTGTGACTCCAGGACTCTAAGGCCTGTCTGGAAGAAAAAAGAAGCAACCTGTAGGCCCATGTGGAGATCGTGAAGGAGGGAAACTTCCCCTGAAGTGGCCCAAAGTGAAGGTGTGGTTCCCAGAGTGGAAATGTGAAGTGGAGAAAAGGACCCTTGTGACGTCGCTGAATGGAGAACTTGCTTGTGCGTCATCAACTAGAATGTCTAGGATAATCACGCCGATTGGGAGAAACCTCTGTTCCTTGCTCCTCTCTCAAAGCTGAGAGAGACCTGGGGAGAGAGGCCCCTGGGCATCACTTCTGCCTACCTGGATCCCTTAATATCCCAGGAGGCGGGAGGATGCTTCCCTGAGAGCCCAGAGGTCACCCAGCAAGGTTAGGGCCAGAAGTGTTCCCAGGGGGAGTGCCATCGTGTCTCAGAGACTTCCTTGGAAGCTATGCTTCTGCTGGCAAGAAGCAGCAGCCGAGCCATCAGAGGCAGGCAGGACTTTGCTGCCTGGAGATCAGATCTCAGTCCTCCCTTGAGGGTCTCCACGGTCAAGTCAGCAGGAGAGCAGGTGCCCTCTCTCTCGGCAGTGGCAGAAACTACAGTGGAGGTGAGACCAGACTGGCAGGGCCGCCAAAGAGCCCACGTGGATGGACCGCCCTTGAAGATCTGTGTGGGCTGTATCTGCTTCAAGTCTGCCTGAAACCCGGGGGTCCAGGAGTCTCTTCCTTATGCCCCAAGGAGCTGACAGACTCTCCAGATGGTTCCCACCTAGGGAGGGTGCCGCGAGGACTAGGCAGTGCTGCTTTATGCACTTGCCATATCCGCTTCACGGATCTGTTTAATCCATCAACTAATGTCGTTAGTGTCACCTGTCATTATGTCGGGCTCCTAGTTTACACTTTAGAAGTCCATTCTCCTTCAAACCAAATGGTTCTTTCTGGCAGACCTGCCCCACCCTATCCTCCTCACCTCCCAGGAAGTCTGGGCTCTTTTTTGCTTTGTGTTCCAAACAAATTCATGCCTCTTACTCATTCCCTTGCTCATTCACCCCTCCTCTCAAAACTTACACTAAAGAGATCTGTGTGCTGGGCCTGTGCCCTTACCAAAGTGCTGTCACCTGCTTATGACACTCCCATGCACCCCCCCCCCCATTACACACAGGAAGGTTTCTTTAGTCTTTCAGTTTCCACGTCTTAATTTCTGAATAGATTCAGATGACCTCCGCTTTTTTTGTGTGCATGTATTTTTTCAACACCAATACATTCTTCATAGGAACTCAATGCCTGCAGAGAGCAGTTCGGAGGCTCTGTTTAGAAAGCACTCCCTGTTCTGAGGTCCTCTGAGGCCCTGGCTGTGCAGATCTGAGAAGGTATGAAGGAGGAGCCACCACGTGCCAGAGGTAGAAAACCAGCGGCCCCACCTTCCCCGCGCACCCCGGGTCTGCTCCCTACCCCATCCTGCCTGCTTCCTGATGTGAAATCTGGGACAGATTTCTGCTTTTAAAGAGCTGCCTGGACAAAGAAGGCGAGTCGGGTTACTCTAGAAACCAAGCAGTCTCGCTGAGGCTACATGACAATTAAGAGCCACGTAGGGAACCGCCTGCTCTAACCAACACTCACCACGTGACTGCGGGAGCACCCGGCATGGAGTGTCCACCGCAGCCCCTGTTGAAACAATGGGGCCCACGAAAGCAATTTAAAG

MSTRG.8326

AAACCAATAGTCTCGCTGTTGGAGGGGGTGATTTGGAGAGCATCCTGGGAAGCAAGTCGTTTCTGGAAAGCCGTGAGGGAAAACAAGGTGGCCCTGATTCCTCCTTCACTGAGAAGGGCAGCTCACACCATGGCAACTGGCTTCCACTGGATGAAGAAGCAAGAGAAGGCACAGCATGCAGAAGTTCCCAGGTCAAGGATTGAACACGTGCCCTAGCAGTGACAACACTGACTGCATCGTTAACCTGCTGAGCCACCAGGGGACTCTACATGAACATTACATTTAACCAGAATGTGTCATGTCTTTCATTATAATTTCAATTGATTTCTCTGTCTCTCTCTCTCTCTCTCTTTTTTTTTTTCCCAGCTGCATCCATGGCATGTGGAAGTTCCCAGGCCAAG

MSTRG.4937

GTGGCCGCCGCCCTGAGCACCCAGGCCGGGAGGAGAGCCGTACGGCCCACAAGCTACCTAGAAAGGGCGGGACGAGAACGTGGGATGCCTGCTCCCCGGACAGTTTCTGAAAGAAGGACCAGAGTAATGCCAGGAATTGCCACATGAAGAGGACCTTCCTGGATCTGTAACTGGGATTAACCAATTCTTGACTTCAGGAGGAAGTTTCAACTATTTCTGAAGATAATGTCAAAACAGGCCCGTGACTCTCCCTTGAAATGGGACAAACAAGCTTCTGGCTTCCCCAGGTCAAGCAGAGAAACACTTATTAGAATGTTGGGTTTCCTCTTTGTTATTCAAACTTACACCCACACACATCCTCCCTAATGAAAAACCTCAACAGATGGAAAGCCTAGGCTCCCAAGCCTGCTGGCTGAGAAGATCACTTCACTAGTCAGTTGGCTGGTACAGAATGGGGGAGGGAGGAGGAATGAGTAGCTAGTACAGGTAATCACTATCTTTTAATACTCCTTACAAATAGAAATTAAAAAAAAAAATCCTCTTCAGCAAGGTTAGCAGACTCTGGGGCCTGGGAAACCAATTTGGCTGATGTGCCTCTGATAGGGAGTAGGCATGGGGTTGCACCAAGGGGAAAGTACTGGCTCTGTATTCTCTCCCTCTCTCTCATTTGAAAGACTGTACAAAGAAATGGTACAAATGGATGGTTAACTTAATGATTCCATCAGAGGTGGATTTTTCTCTGTCAGGTCCCTGGATGAGGCTGAGGTCTGAGAGCCAGAAGGTAGGAATGTGTAATCGCCCTTTCTTGCCACTTTTCCAGGGGCCAGGACTACACTTAAGCCCTTTTGCTCTGCAACTTCCCCATGCCCCTGGCTAGCTCAGCTGCAACTCCTCCTTCCACTCCAGGCTGAGTCCTGAGTTTCTCCTGGTAGCCAGGGCAAGCTTCCCTCTGCCTGAATCTCCCTCCCCCTCCACCCCGCCTCCCATCTTCCAGCAGGCCGTTCCAGCTATCTCCCCTCTACCCTGCTTTAATTCAAGTCTGATTGTCTCCACCGAACAACCAGGGGCTCCCAAATGAGGGATGCCACCTCTCATCCTTGGGCCTGGGCTTGAGGCCCTGACCCCTTAAGGACCCAGGCGGATCCCCTACAGCCTCCCTGTGGGAAAAAGGCAGAGCTGGCCCAGGCCGGAGCC
